# The Downregulation of Placental Lumican Promotes the Progression of Preeclampsia

**DOI:** 10.1007/s43032-021-00660-w

**Published:** 2021-07-06

**Authors:** Chao Liu, Yulian Hu, Zhongying Wang, Hua Pan, Yan Ren, Xiao Li, Zhiqiang Liu, Huijie Gao

**Affiliations:** 1 College of Pharmacy, Jining Medical University, Rizhao, Shandong China; 2 Department of Obstetrics, People’s Hospital of Rizhao, Rizhao, Shandong China; 3 Department of Gynecology, People’s Hospital of Rizhao, Rizhao, Shandong China

**Keywords:** Lumican, Preeclampsia, Trophoblast, Small leucine-rich proteoglycan, P53, Bcl-2

## Abstract

Multiple pieces of evidence illustrate that impaired trophoblast function results in preeclampsia (PE), and migration/invasion of human trophoblast cells is stringently regulated by extracellular matrix (ECM) components. Many studies have indicated abnormal expressions of placental ECM components are associated with preeclampsia. However, the change and influence of lumican, a vital member of extracellular matrix (ECM) molecules, on trophoblast cells during preeclampsia remain unclear. This study examines the possibility that the roles of lumican in trophoblast cells contribute to PE. To address this issue, the expression of lumican in human placental tissues was observed using immunohistochemistry, fluorescence quantitative PCR, and Western blot technology. After the HTR-8/SVneo cell line was transfected with pcDNA3.1-human lumican, pGPU6-human lumican shRNA, and their negative controls, the impact of lumican on the HTR-8/SVneo cell line was investigated. Lumican was expressed in human placental tissues. Compared with the control group, its expression was significantly lower in PE placentas. Lumican downregulation inhibited cell proliferation significantly and reduced Bcl-2 expression, but increased P53 expression. These results indicate that the downregulation of placental lumican may drive PE development via promoting the downregulation of Bcl-2 expression and upregulation of P53.

## Introduction

Preeclampsia (PE) is a dangerous clinical syndrome of pregnancy that develops after week 20 of gestation [1–5]. It is typically manifested as new-onset maternal hypertension (≥140/90 mmHg) and proteinuria [1–3]. PE affects approximately 4.2% of pregnancies in China and 2–10% globally and is a leading contributor to maternal and perinatal mortality and morbidity, especially in developing countries [4, 5]. Despite great advances in perinatal medicine, PE cannot be prevented, and the best therapy for it is still the parturition of the placenta; this suggests that the placenta plays an important part in the pathogenesis of PE [2, 6, 7].

Multiple pieces of evidence illustrate that preeclampsia results from the presence of a placenta [2]. The placenta is a highly regulated invasive organ derived from extraembryonic tissues, and its invasion of the uterus originates from trophoblast proliferation, migration, and invasiveness. Impaired trophoblast function results in abnormal placentation, which causes pregnancy-associated clinical syndromes such as PE [6–11]. Among the many factors affecting trophoblast function, increasing evidence has shown that cell–extracellular matrix (ECM) interactions play a fundamental role in the proliferation and differentiation of, and invasion by, trophoblast cells [12, 13]. Migration/invasion of human trophoblast cells is stringently regulated by extracellular matrix (ECM) components, and many studies have indicated abnormal expressions of placental ECM components are associated with preeclampsia. The ECM is a complex meshwork of material that surrounds cells, comprising glycoproteins, proteoglycans, and glycosaminoglycans. Lumican is a major proteoglycan of the cornea, where it was first identified [14, 15]; its gene occurs on chromosome 12q21.3-q22, and the protein has 338 amino acids. It is highly expressed in the human cornea, skin, kidney, heart, pancreas, and placenta [16–22]. In the cornea and skin, lumican promotes cell migration and proliferation during tissue repair. There have been conflicting reports about its role in tumor progression [23–29].

Nonetheless, it remains unclear how lumican, or the composition of the ECM generally, changes in the placenta; further, it is unclear how these changes influence trophoblast cells during preeclampsia. Therefore, we hypothesized that the interaction of lumican with trophoblast cells might contribute to PE. To test this, we compared the expression of the mRNA and protein of placental lumican between PE patients and controls and assessed the function of lumican in trophoblast cells.

## Materials and Methods

### Patients and Tissue Samples

The present study was consistent with the Declaration of Helsinki and received approval from the Ethical Committees of Jining Medical University (2019-YX-010, March 2019 to December 2023). All patients provided written informed consent. Fifty seven control subjects and 45 PE patients from the Rizhao People’s Hospital were recruited between June 2019 and January 2021. The clinical characteristics of each subject that were recorded include maternal age, gestational age (at admission and at delivery), body weight, and blood pressure (systolic and diastolic). Patients’ clinical characteristics are detailed in Table 1.

**Table 1:** The clinical characteristics of PE and control groups

Characteristics	PE (N=45)	Control (N=57)	t	*p* value
Maternal age (years)	30.09±3.29	30.14±2.96	0.083	0.934
Times of gravidity	2.07±0.69	2.02±0.64	–0.372	0.71
Age of menarche (years)	14.02±0.75	14.07±0.70	–0.331	0.741
Gestational age at delivery (weeks)	36.20±1.84	39.26±0.77	11.378	<0.001
Fetal birth weight (kg)	2.62±0.39	3.45±0.29	12.25	<0.001
Systolic blood pressure (mmHg)	165.33±8.53	116.54±8.48	70.067	<0.001
Diastolic blood pressure (mmHg)	105.71±8.71	74.60±5.07	–25.571	<0.001

According to the guidelines of the American College of Obstetricians and Gynecologists, preeclampsia was identified based on new-onset hypertension (≥140/90 mmHg) and proteinuria (300 mg or more per 24-h urine collection or protein to creatinine ratio of 0.3 mg/dL or more or dipstick reading of 2+) that developed after week 20 of gestation [30]. None of the PE patients had past medical history of systemic diseases such as hypertension, altered renal function, heart disease, hepatic diseases, diabetes mellitus, or of receiving blood transfusion. Subjects with the following criteria were excluded as controls: (1) maternal age <26 years; (2) gestational age at admission <30 weeks; (3) past medical history of systemic diseases including hypertension, altered renal function, heart disease, hepatic diseases, diabetes mellitus, or of receiving blood transfusion; and (4) obstetric complications.

Four full-depth biopsies, of tissue without bleeding or necrosis, were collected from the typical biopsy area between the placental periphery (2 cm from the placental border) and the peri-insertion site (2 cm from the spinal cord insertion point). Tissue contaminated with attached decidua was excluded. Tissues were immediately divided into pieces and frozen below –80°C until RNA and protein extraction. The sampling procedure was completed within 30 min after placental delivery.

### Immunohistochemistry

Paraffin-embedded tissue sections were immunostained using the SABC (Goat IgG)-POD Kit. After deparaffinization, hydrogen peroxide (3%) was used to quench endogenous peroxidase activity for 30 min. After another 20 minutes of blocking using BSA, the sections were incubated for 24 h with the anti-lumican antibody at 4°C (1:1000, using Primary Antibody dilution buffer). The sections were then incubated for 30 min with the anti-goat IgG (Bio-Rabbit) at 37°C (1:100, using Primary Antibody dilution buffer) and with SABC-POD for 30 min at 37°C (1:100, using Primary Antibody dilution buffer). Finally, diaminobenzidine-tetrahydrochloride (DAB) was used to visualize the antibody binding site, and Mayer’s hematoxylin was used to visualize the nuclei.

### Cell Culture

The immortalized HTR-8/SVneo cell line (Fenghui Shengwu Biotech Co., Ltd., Wuhan, Hubei, China) was cultured in high-glucose Dulbecco’s Modified Eagle’s medium (DMEM) containing 10% fetal bovine serum (FBS) from Hyclone, streptomycin (100 μg/mL), and penicillin (100 IU/mL), in a humidified 5% CO_2_ incubator at 37°C. The cells were passaged at 70–90% confluence.

### Vector Construction and Transfection of Human Lumican cDNA

The cell expression construct (pGPU6-human lumican shRNA and pcDNA3.1-human lumican) was purchased from Fenghui Shengwu Co., Ltd. The cells were cultured in high-glucose DMEM containing 10% FBS, but with no streptomycin or penicillin. Subsequently, the cells were grown to 60% confluence and then transfected with pcDNA3.1-human lumican or pGPU6-human lumican shRNA using the ExFect2000 Transfection Reagent, following the manufacturer’s instructions. After 24–72 h of culture, the cells were used for Cell Counting Kit-8 (CCK-8) assay or subsequent RNA and protein extraction.

### Cell Counting Kit-8 (CCK-8) Assay

The cell proliferation of the HTR-8/SVneo cell line was assessed via CCK-8 assays (Vazyme Biotech). After 48 h of transfection, the mixed liquor containing 90 μl of DMEM and 10 μl of CCK-8 solution was added to each well, into which 5000 cells were placed, followed by 2-h incubation. Absorbance (*A*) at 450 nm was then assessed via a SYNERGY H1 Hybrid Multi-Mode Microplate Reader (BioTek Instruments, Inc, Winooski, Vermont, USA).

### TUNEL Assay

Cells from appropriate treatment groups were resuspended at 1 x 10^8^ cells/L in a 3.5-cm Petri dish containing cover slides and were incubated for 24 h at 37°C. Media was then replaced, followed by an additional 72-h incubation after which cell morphology was imaged. A TUNEL BrightRed

Apoptosis Detection Kit was then used to stain cells based on provided directions, and cells were imaged via fluorescent microscope at 620 nm and 460nm.

### Wound Healing Assay

HTR-8/SVneo cells were serum-starved and transfected with appropriate plasmids prior to this assay to minimize the impact of proliferation on invasive activity. Cellular monolayers were then scratched using a sterile 100-μL pipette to generate a wound. Cells were then washed thrice with PBS, and cells were incubated for 24 h. Images of cells at 0 and 24 h post-wound generation were compared to assess invasive activity.

### RNA Extraction and Quantitative Real-Time PCR

Total RNA from the human placental tissues and HTR-8/SVneo cells was extracted using TRIzol reagent (Invitrogen) as per the manufacturer’s instructions, after which a NanoDrop 2000c (Thermo Scientific) instrument was used to assess total RNA quantity and quality. Then, 1 ug of total RNA was used to prepare cDNA using a ThermoScript Reverse transcription kit (Vazyme Biotech). Real-time quantitative reverse transcription PCR (qPCR) reaction was conducted in a final volume of 20 μl, containing 10 μl Maxima SYBR Green, 1 μl cDNA, 1 μl each of the forward and reverse primers, and DNase-free H_2_O. In addition, the housekeeping gene β-actin was assessed as a normalization control. The primer sequences are listed in Table 2. A CFX96 real-time system (Bio-Rad, CA, USA) was used to perform the qPCR reactions, with the following thermocycler settings: denaturing at 94°C for 5 min and 38 cycles of 94°C for 45 s, 55°C for 45 s, and 72°C for 90 s. These qPCR reactions were conducted in triplicate, and relative gene expression was assessed via the 2^−ΔΔCt^ approach.

**Table 2:** qPCR primers used in the present study

Gene	Primer sequences	
Lumican	Forward Reverse	CTTCAATCAGATAGCCAGACTGC AGCCAGTTCGTTGTGAGATAAAC
P53	Forward	GAGGTTGGCTCTGACTGTACC
	Reverse	TCCGTCCCAGTAGATTACCAC
Bcl-2	Forward	GGTGGGGTCATGTGTGTGG
	Reverse	CGGTTCAGGTACTCAGTCATCC
β-actin	Forward	CATGTACGTTGCTATCCAGGC
	Reverse	CTCCTTAATGTCACGCACGAT

### Western Blotting

The extraction of total protein from the HTR-8/SVneo cells and human placental tissue was performed using RIPA buffer with protease and phosphatase inhibitors, according to the manufacturer’s instructions. The protein concentration was calculated using the BCA protein assay kit. Thereafter, equal amounts of each protein sample were separated via 10% SDS-PAGE and transferred to PVDF membranes. The membranes were then immersed in blocking fluid, washed, and incubated for 16 h at 4°C with the suitable primary antibodies. The primary antibodies included lumican (AF2846; R&D Systems), tubulin (BM3877; Boster), P53 (BM0101; Boster), and Bcl-2 (BM0200; Boster). After washing, the membranes were incubated with the secondary antibody. Finally, protein bands were assessed with an ECL chemiluminescence detection kit (Millipore). Protein expression was calculated using an iBright FL1000 Imaging System (Invitrogen, Thermo Fisher Scientific).

### Statistical Analysis

Data for all experiments are given as the mean ± SD and were evaluated via a two-tailed Student’s *t*-test or one-way ANOVA, with Tukey’s post-hoc test as appropriate, using SPSS 18.0 (IBM, Chicago, IL), with *P* < 0.05 as the significance threshold. All experiments were repeated in triplicate.

## Results

### Lumican Expression Was Downregulated in PE Placentas

**Fig. 1:**
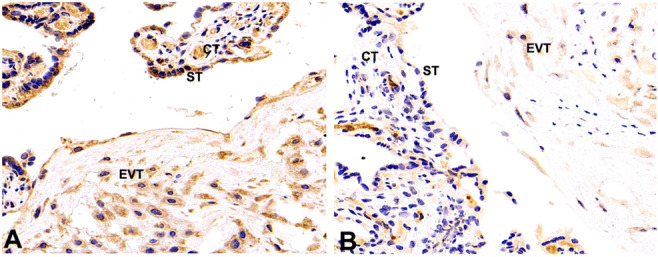
The expression of lumican was assessed via IHC in PE placentas (**B**) and control group (**A**). The positive cells (ST, CT, EVT) were brown in the cytoplasm, and darker brown cells were found in the control group. ST, syncytiotrophoblast; CT, cytotrophoblast; EVT, extravillous trophoblasts

**Fig. 2:**
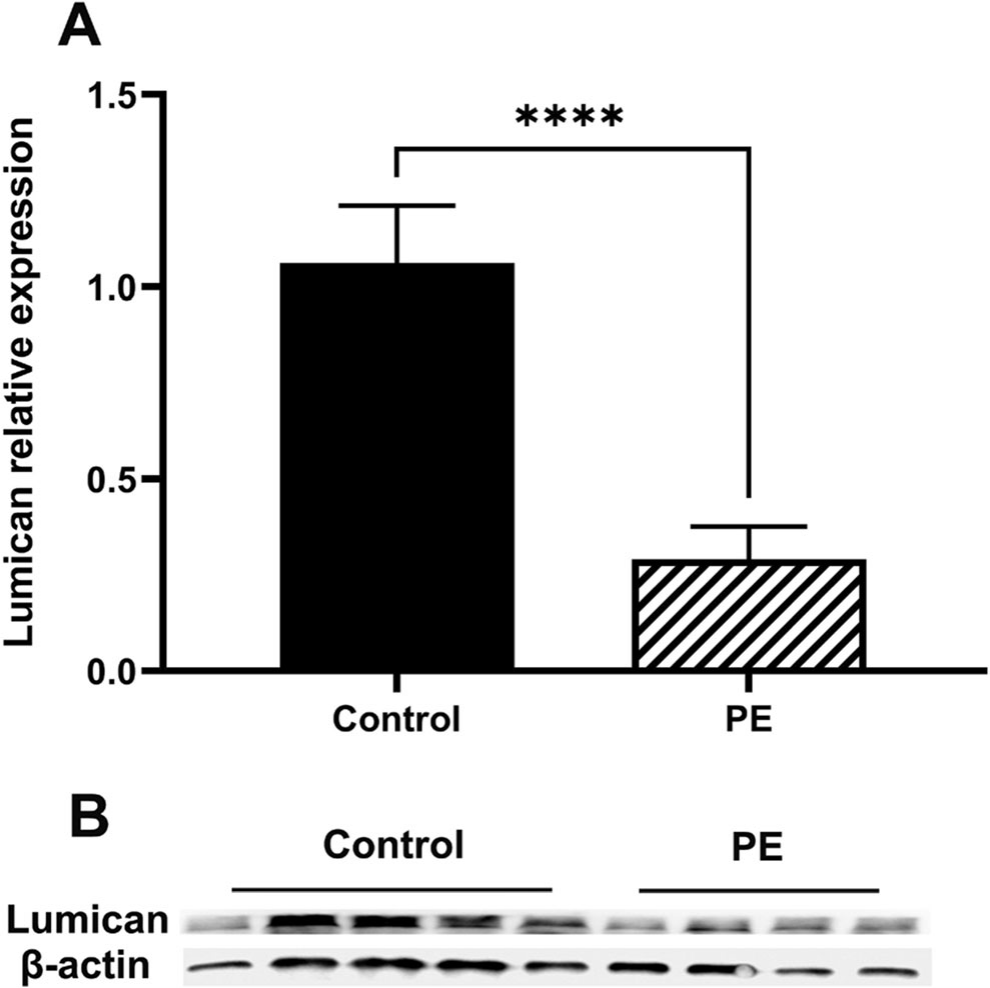
The expression of lumican in human placentas. **A** Lumican mRNA expression was assessed via qPCR in PE placentas (0.29±0.08) and control group (1.06±0.15). **B** Lumican expression was assessed via Western blotting in PE placentas (1.49±0.81) and control group (3.09±1.11). ****p<0.0001

To explore the role of lumican in PE, lumican expression in placentas was examined first via immunohistochemical staining. Lumican was widely expressed in trophoblast cells. The positive signal of lumican (brown staining) was lower in PE placentas (Fig. 1B) than in the control group (Fig. 1A). Consistent with this, placental lumican expression was 3.6-fold higher in the control than in the PE group, at the mRNA level (Fig. 2A). Similarly, protein-level lumican dysregulation was detected in PE placentas (Fig. 2B).

### The Impact of Lumican on Trophoblast Cells

**Fig. 3:**
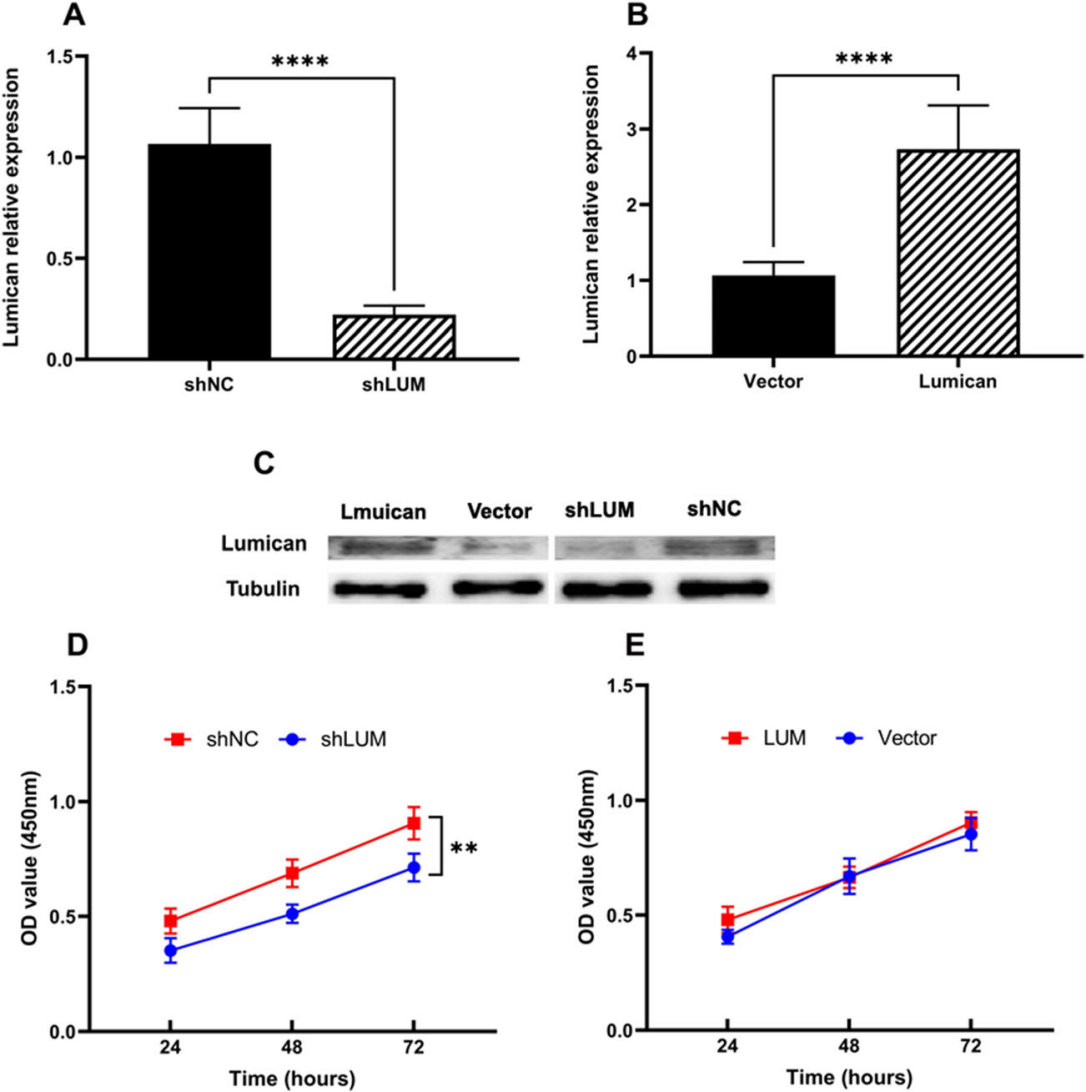
Lumican influences PDAC cell growth. **A** Lumican mRNA expression was assessed in HTR-8/SVneo cells following transfection with pGPU6-human lumican shRNA (shLUM, 0.22±0.04) and their negative controls (shNC, 1.06±0.18). **B** Lumican mRNA expression was assessed in HTR-8/SVneo cells following transfection with pcDNA3.1-human lumican (lumican, 2.73±0.57) and their negative controls (vector, 1.07±0.17). **C** Lumican protein-level expression was assessed in HTR-8/SVneo cells following transfection with pcDNA3.1-human lumican (lumican), pGPU6-human lumican shRNA (shLUM), and with their negative controls (vector or shNC). **D** and **E** The proliferation of HTR-8/SVneo cells was assessed following transfection with pcDNA3.1-human lumican (lumican), pGPU6-human lumican shRNA (shLUM), and with their negative controls (vector or shNC) was assessed. ****p<0.0001

**Fig. 4:**
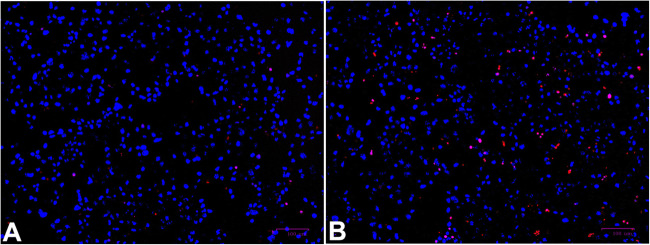
HTR-8/SVneo cellular apoptosis following pGPU6-human lumican shRNA (**B**) or control construct (**A**) transfection was assessed via TUNEL staining

**Fig. 5:**
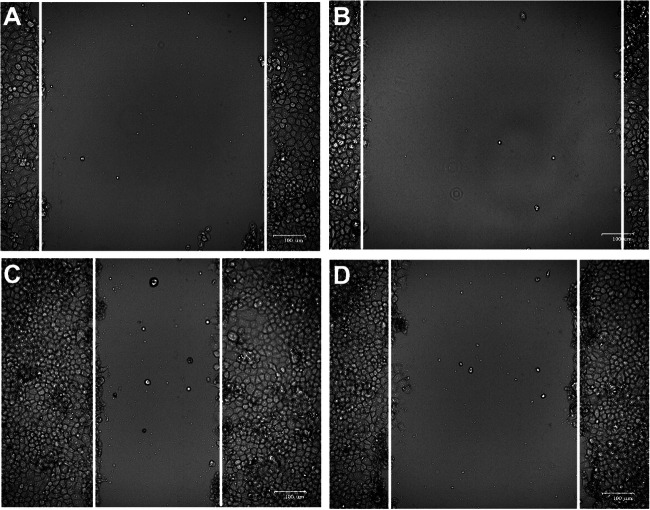
HTR-8/SVneo cell migratory activity following pGPU6-human lumican shRNA (**B**, **D**) or control construct (**A**, **C**) transfection was assessed via wound healing assays. (**A**, **B**) Images of cells at 0 h post-wound generation. (**C**, **D**) Images of cells at 24 h post-wound generation

To explore the role of lumican in PE, the impact of lumican on trophoblast cells was investigated by transfecting HTR-8/SVneo cells with pcDNA3.1-human lumican, pGPU6-human lumican shRNA, and with their negative controls. Lumican mRNA and protein expression in HTR-8/SVneo cells was assessed after 72 h, via qPCR and Western blotting, revealing that lumican levels were significantly lower in cells transfected with pGPU6-human lumican shRNA and significantly higher in those transfected with pcDNA3.1-human lumican than in their respective negative control groups (Fig. 3A–C). The cell proliferation of HTR-8/SVneo was examined using CCK-8 assays. This revealed that lumican downregulation significantly inhibited the proliferation of HTR-8/SVneo cell (Fig. 3D), whereas its overexpression did not (Fig. 3E). Subsequent TUNEL staining revealed that lumican downregulation markedly increased HTR-8/SVneo cellular apoptosis (Fig. 4). Wound healing assays also revealed that lumican knockdown was associated with a marked decline of HTR-8/SVneo cell migratory activity relative to cells transfected with a negative control construct (Fig. 5).

### Lumican Induces Changes in P53 and Bcl-2 in Trophoblast Cells

**Fig. 6:**
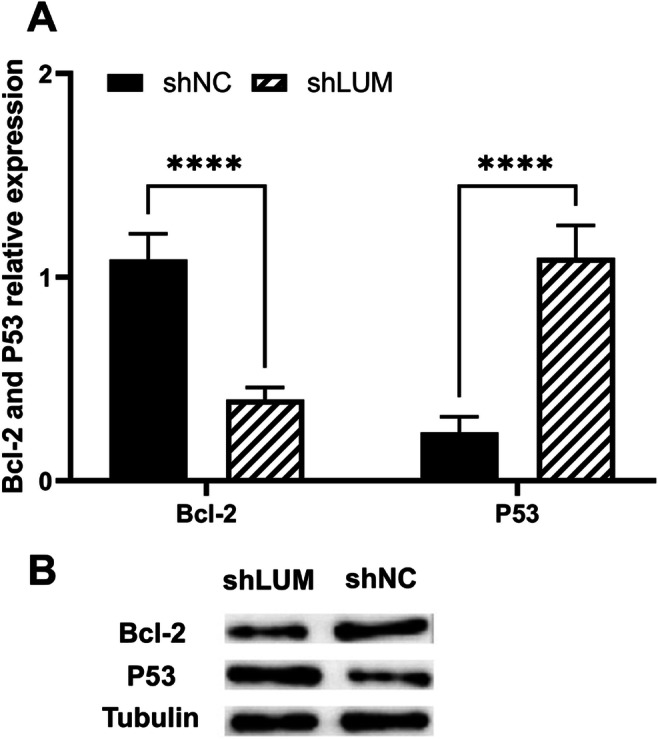
Lumican influences P53 and Bcl-2 expression in HTR-8/SVneo cells. **A** P53and Bcl-2 expression was assessed via qPCR in HTR-8/SVneo cells that had been transfected with the pGPU6-human lumican shRNA (P53, 1.10±0.16; Bcl-2, 0.24±0.08) or control constructs (P53, 0.4±0.06; Bcl-2, 1.09±0.13). **B** P53and Bcl-2 protein-level expression was evaluated in HTR-8/SVneo cells transfected with pGPU6-human lumican shRNA (shLUM) or control (shNC) constructs. ****p<0.0001

To explore the pathway regulated by lumican in trophoblast cells, we analyzed some apoptosis-related proteins involved in trophoblast cell proliferation. qRT-PCR and Western blot analysis revealed that transfection of HTR-8/SVneo cells with pGPU6-human lumican shRNA reduced the expression of Bcl-2 in these cells and increased P53 expression (Fig. 6). This indicates that lumican downregulation leads to downregulation of Bcl-2 and upregulation of P53, which inhibits cell proliferation.

## Discussion

Despite the great advances that have been made in perinatal medicine research, PE remains a dangerous and unpreventable disease that is highly associated with maternal and perinatal mortality and morbidity [1–5]. An increasing body of literature suggests that impaired trophoblast function results in abnormal placentation, which causes pregnancy-associated clinical syndromes such as PE [8–12].

Small leucine-rich proteoglycan (SLRP) family proteins, which are highly expressed in the placenta, are major components of the ECM and are thought to play important roles in multiple processes, including cell adhesion and migration, angiogenesis, and embryonic development [31–35]. Based on the importance of cell–ECM interactions in trophoblast cell proliferation, differentiation, and invasiveness [13, 14], it is likely that the SLRPs play important roles in the pathogenesis of PE by affecting trophoblast function. Nonetheless, few papers have examined SLRP changes in the placenta and their influence on trophoblast cells during preeclampsia. Using real-time PCR and Western blot analysis, Chui et al. found that biglycan and decorin mRNA and protein expression were lower in PE placentas than in control placenta [35]. Peeyush et al. demonstrated that decorin is likely to negatively regulate trophoblast function [36].

Lumican, a class II SLRP, has a 40 kDa core protein comprising four major domains. Lumican expression differs among human tissues [18–23]. It is highly expressed in the cornea, where it promotes corneal epithelial wound healing, by inducing keratinocyte proliferation and migration [14, 37–40]. Its expression in tumors has been observed in various studies: in most of these, it has been found to limit tumor invasion via various mechanisms [23–27]. In contrast, high lumican expression was associated with reduced survival and higher rates of metastasis in advanced colorectal cancer and gastric cancer [28, 29]. Overall, its effects on cell function vary greatly and are highly tissue-specific.

Although lumican is highly expressed in the placenta [22], its expression and effects on trophoblast cells remain unclear in PE. Our study examined, for the first time, lumican expression in the placentas of PE patients and controls. The expression of its mRNA and protein was significantly lower in PE placentas than in control placentas. This finding, which is in line with those of Chui et al. [35], suggests that reduced lumican expression may contribute to PE. Accordingly, it is urgent to elucidate the effects of lumican in trophoblast cells. To examine this, we transfected pcDNA3.1-human lumican, shR-human lumican, and their negative controls into HTR-8/SVneo trophoblast cells to alter their lumican expression. CCK-8 assay revealed that lumican downregulation significantly inhibited their cell proliferation, whereas its overexpression did not. Considering the reduced lumican expression in the PE placentas, it is likely that lumican downregulation, rather than overexpression, is the major factor affecting trophoblast cell function. Many studies have shown that Bcl-2 is a classic antiapoptotic protein and that P53 is a typical tumor suppressor gene. Our findings indicate that lumican downregulation leads to downregulation of Bcl-2 and upregulation of P53. This provides an effective potential route for examining pathway regulation by lumican in HTR-8/SVneo cells.

No prior studies have evaluated the functional importance of lumican in PE placentas. We found that lumican expression was significantly lower in PE placentas than in healthy placentas. Furthermore, downregulation of lumican significantly inhibited HTR-8/SVneo cell proliferation, via a mechanism associated with downregulation of Bcl-2 and upregulation of P53, suggesting that this may be a mechanism whereby this proteoglycan influences PE progression. Future preclinical studies about how this proteoglycan affects PE patient outcomes will enable us to better understand its functional role in vivo and will assist in improving PE therapy and prognosis. In addition, further work is needed to verify our experimental results. For example, although the gestational age at delivery of all subjects in our study was on the third trimester, difference in gestational week between PE and control does exist, and the influence of advancing gestational age on the expression of lumican in the placenta is an issue not to be ignored. Although collecting enough PE patients and gestational age-matched control subjects will take a large amount of time, it is worthwhile to address this issue in the future study.
